# Rubeosis faciei diabeticorum is not associated with oxidative stress and skin autofluorescence^☆^^☆☆^

**DOI:** 10.1016/j.abd.2019.09.016

**Published:** 2019-09-30

**Authors:** Aleksejs Zavorins, Alise Silova, Julija Voicehovska, Janis Kisis

**Affiliations:** a Department of Infectology and Dermatology, Riga Stradins University, Riga, Latvia; b Scientific Laboratory of Biochemistry, Riga Stradins University, Riga, Latvia; c Department of Internal Diseases, Riga Stradins University, Riga, Latvia

**Keywords:** Diabetic angiopathies, Erythema, Face, Glycation end products, advanced, Oxidative stress, Skin manifestations

## Abstract

**Background:**

Rubeosis faciei diabeticorum is a persistent facial erythema in patients with diabetes mellitus. The actual pathogenesis has not been studied. However, it is speculated to be a cutaneous diabetic microangiopathy.

**Objective:**

Examine the correlation between the severity of facial erythema and the possible causes of microvascular diabetic complications, namely oxidative stress, hyperglycemia, and cutaneous accumulation of advanced glycation end-products .

**Methods:**

Patients diagnosed with Type 2 diabetes mellitus (*n* = 32) were enrolled in the study. The facial erythema index was measured using the Mexameter MX18; cutaneous accumulation of advanced glycation end-products was estimated by measuring skin auto fluorescence with the AGE Reader (DiagnOptics Technologies B.V. – Groningen, Netherlands). Glycated haemoglobin, total antioxidant status, and malondialdehyde were measured in blood by TBARS assay. The correlation between the selected variables was assessed by Spearman's rank test; *p* ≤ 0.05 was considered statistically significant.

**Results:**

There was a statistically significant correlation between total antioxidant status and the facial erythema index (*ρ* = 0.398, *p* = 0.024). Malondialdehyde, skin autofluorescence, glycated haemoglobin, body mass index, duration of diabetes, and age did not demonstrate statistically significant correlation with the facial erythema index.

**Study limitations:**

This is an observational study. Elevation of total antioxidant status could have been caused by several factors that might have also influenced the development of rubeosis faciei, including hyperbilirubinemia and hyperuricemia.

**Conclusions:**

The results contradicted expectations. Total antioxidant status correlated positively with facial erythema index; however, there was no correlation with oxidative stress and skin autofluorescence. Further investigations should be conducted to reveal the cause of total antioxidant status elevation in patients with rubeosis faciei.

## Introduction

Diabetes mellitus (DM) is a disease characterized by persistent hyperglycemia, either due to an autoimmune destruction of pancreatic β-cells and impaired production of insulin (Type 1 DM), or due to the failure of peripheral tissues to respond properly to insulin (Type 2 DM). DM can also be caused by a pancreatic disease or trauma, as well as pregnancy, certain genetic defects, medication, and endocrinopathies.^1^ Type 2 DM accounts for up to 90% of all diabetes cases; it is strongly associated with obesity and a genetic predisposition.^1^, ^2^ The global prevalence of DM was estimated to be 8.8% in 2017.^2^ Diabetes is associated with the development of macrovascular and microvascular complications, namely retinopathy, neuropathy, and nephropathy. The prevalence of retinopathy amongst patients with diabetes is estimated at 34.6%, with approximately one third developing a vision-threatening diabetic retinopathy.^3^ Diabetic neuropathy and nephropathy are common causes of lower limb amputations and end-stage renal disease, respectively.^4^, ^5^ The development of microvascular complications is related to chronic hyperglycemia, as demonstrated in the UK Prospective Diabetes Study (UKPDS) and the Diabetes Control and Complications Trial/Epidemiology of Diabetes Interventions and Complications Study (DCCT/EDIC).^6^, ^7^, ^8^ Therefore, the role of early diagnostics and treatment of diabetes mellitus cannot be understated.

Rubeosis faciei diabeticorum is a cutaneous manifestation of diabetes mellitus. It is characterized by a diffuse, persistent facial erythema that is more common amongst diabetic patients. Increase in number and diameter of superficial venules in the cheeks of diabetic patients has been described histologically.^9^ Due to the lack of recent investigations in this field, the exact prevalence and pathogenesis of rubeosis diabeticorum is not known. However, it has been speculated to be a form of a diabetic microangiopathy, and therefore could be proposed as a potential marker for other diabetic microvascular complications. No articles that have actually examined this association have been found.^9^, ^10^, ^11^

Oxidative stress is thought to be a crucial link between hyperglycemia and the development of diabetic microangiopathies.^12^, ^13^ Oxidative stress is defined as an excessive production of reactive oxygen species (ROS), such as superoxide, hydroxyl, and hydrogen peroxide, and the insufficient capacity of the antioxidant system to combat it.^14^ Brownlee has postulated that hyperglycemia causes overproduction of superoxide by the mitochondrial electron-transport chain, which in turn activates four pathways that promote microvascular damage in diabetes: an increased flux of glucose through the polyol pathway, enhanced production of glucose-derived advanced glycation end-products (AGEs), induction of protein kinase-C activity (PKC), and upregulation of hexosamine pathway activity.^13^, ^14^, ^15^, ^16^, ^17^, ^18^

AGEs are products of a non-enzymatic glycation or Maillard reaction, which is enhanced in the event of hyperglycemia and oxidative stress.^19^ AGEs form abnormally stable cross-links with collagen and alter intracellular proteins, including transcription regulating factors. AGE-related glycation of the mitochondrial respiratory chain proteins promotes further production of ROS.^17^ Activation of receptors for AGEs (RAGEs) induces expression of the pro-inflammatory nuclear factor – κB (NF-κB).^20^ Vascular endothelial growth factor (VEGF) is a crucial angiogenic signal protein that promotes proliferation and migration of endothelial cells and increases vessel permeability.^20^ NF-κB enhances VEGF expression and triggers retinal neovascularization, which is a feature of proliferative diabetic retinopathy.^21^ Changes in the conjunctival microvasculature have also been noted in patients with diabetic retinopathy.^22^, ^23^

The aforementioned processes could also promote angiogenesis in the facial skin, which is clinically apparent as rubeosis faciei diabeticorum. Therefore, the goal of this study was to examine the possible correlation between oxidative stress, hyperglycemia, cutaneous accumulation of AGEs, and the degree of rubeosis faciei.

## Methods

### Study population

Patients diagnosed with Type 2 diabetes mellitus (*n* = 32) according to the diagnostic criteria of the World Health organization (WHO, 2011) were enrolled in the study.^1^ A structured interview was conducted to obtain data on the duration of diabetes, smoking status, medication usage, and presence of hypertension. Individuals who had history of cardiovascular events, polycythemia, as well as clinical signs of an acute illness, actinic damage, or an inflammatory facial skin conditions such as seborrheic dermatitis, lupus erythematosus, papulopustular rosacea, eczema, psoriasis, and dermatomyositis were excluded from further participation. Physical examination was performed to assess the body mass index. The measurements were conducted during winter and early springtime. All patients were phototypes I–II according to Fitzpatrick, with no signs of skin tan. The acquired data is summarized in Table 1. Written informed consent was obtained from all patients. The study protocol was approved by the Ethics Committee of Riga Stradins University (approval No. 79/25.01.2018).

**Table 1:** Characteristics of type 2 diabetes mellitus patients

	Mean (SD)
Age, years	59.56 (14.85)
Duration of diabetes, years	9.90 (5.86)
BMI, kg/m^2^	32.68 (5.37)
HbA1c, %	7.60 (1.78)

	Quantity (%)
Gender (males/females)	13 (40.6)/19 (59.4)
Active smokers	5 (15.6)
Arterial hypertension	23 (71.9)

BMI, body mass index.

### Erythema assessment

Rubeosis faciei or facial erythema in diabetic patients and the control group was assessed using the Mexamater MX18 (Courage & Khazaka electronic GmbH – Cologne, Germany). It is a colorimetric device that emits specific wavelengths and analyses the light that is reflected from the skin. This allows calculation of the amount of light absorbed by haemoglobin and presents it as an erythema index (EI) in arbitrary erythema units (0–999 AEU).^24^ Patients were allowed to rest for 30 min inside a room with a stable temperature of 23 °C before the measurement took place. The EI was measured in five places: left and right cheek, forehead, nose, and chin. The mean EI value for each patient was calculated for further analysis.

### Skin autofluorescence measurement

AGE levels in the skin were estimated non-invasively by means of skin autofluorescence measurement. The measurements were conducted using the AGE Reader (DiagnOptics Technologies B.V. – Groningen, Netherlands) in a room at 23 °C after an overnight fasting. The AGE Reader utilizes the fact that certain AGEs have fluorescent properties. A cutaneous area of 4 cm^2^ is excited by the ultraviolet light. Subsequently, an optical spectrometer detects light emitted by the skin. The software estimates the relative concentration of AGEs in the skin and presents it using AEU. The measurements were made on the right, inner forearm approximately 5 cm distally from the cubital fossa.

### Laboratory analysis

Blood samples were drawn from the antecubital vein of the diabetic patients after overnight fasting.

Total antioxidant status (TAS) was measured by automated spectrophotometry with the RX Daytona Analyzer (Randox Laboratories, Ltd. – United Kingdom) in the plasma of diabetic patients. The RANDOX TAS kit (Cat. No. NX 2332; Randox Laboratories, Ltd. – United Kingdom) was used according to the manufacturer's instructions. Plasma was obtained from tubes that contained lithium heparin. Samples were centrifuged at 3500 rpm and stored at −20 °C until analysis. ABTS® (2.2′-Azino-di-[3-ethylbenzthiazoline sulphonate]) is incubated with metmyoglobin and H_2_O_2_ to produce the radical cation ABTS®*^+^, which is characterized by blue-green colour measured at 600 nm. Antioxidants in patients’ plasma decrease formation of this colour, proportionally to their concentration.^25^, ^26^

Malondialdehyde (MDA) was measured by spectrophotometry with the Sunrise absorbance microplate reader (TECAN – Switzerland) in the plasma of diabetic patients. The OxiSelect™ thiobarbituric acid reactive substances (TBARS) assay (Cat No. STA-330, CellBiolabs, Inc, United States) was used according to the manufacturer's instructions. Plasma was obtained from tubes that contained EDTA. Samples were centrifuged at 3500 rpm and stored at −80 °C until analysis. Samples were reacted with TBA at 95 °C. After a brief incubation, results are obtained spectrophotometrically. To evaluate the MDA content in patients’ plasma, the results are compared to the standard curve for MDA.^27^

Glycated haemoglobin (HbA1c) was measured using turbidimetric inhibition immunoassay (TINIA) in hemolysate with the Cobas Integra analyzer (Roche – Mannheim, Germany).

### Statistical analysis

The data was analyzed using SPSS (v. 23) software. The correlation between the selected variables was assessed by Spearman's rank test because of the small sample size and non-normal data distribution on several occasions, as observed by the Shapiro–Wilk test; *p*-values ≤ 0.05 were considered statistically significant.

## Results

**Figure 1:**
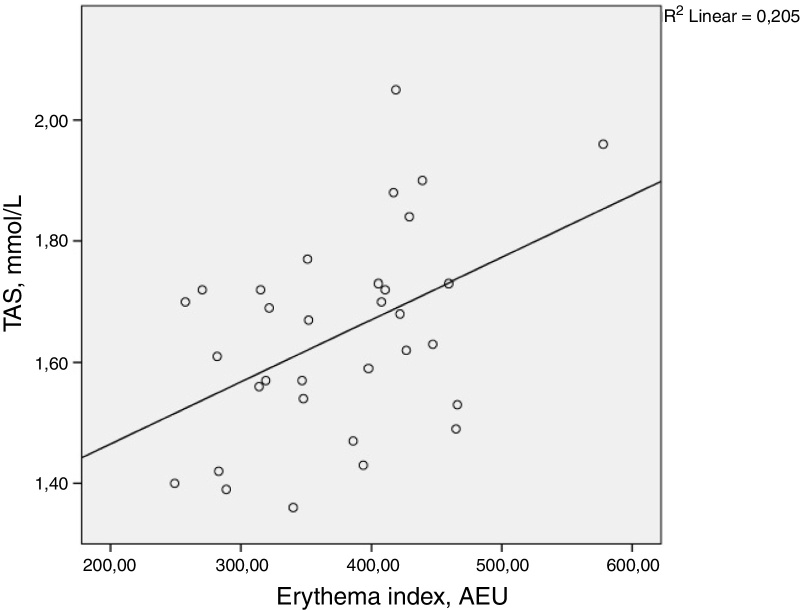
There was a statistically significant correlation between total antioxidant stress (TAS) and the facial erythema index (*ρ* = 0.398, *p* = 0.024). AEU, arbitrary erythema units.

There was a statistically significant correlation between TAS and facial erythema index (*ρ* = 0.398, *p* = 0.024). MDA, skin autofluorescence, glycated haemoglobin levels, BMI, duration of diabetes, and age did not demonstrate statistically significant correlation with the facial erythema index. The results are summarized in Table 2 and Fig. 1.

**Table 2:** Spearman rank correlation between the facial erythema and several measured parameters. *p*-Values ≤ 0.05 are considered statistically significant and are written in bold

	Facial erythema index, AEU	
	Spearman's *ρ*	*p*-Value
TAS, mmoL/L	0.398	**0.024**
MDA, mmoL/L	0.302	0.093
Skin autofluorescence, AU	0.157	0.389
HbA1c, %	−0.117	0.525
Age, years	−0.014	0.940
Duration of diabetes, years	−0.127	0.488
BMI, kg/m^2^	0.338	0.058

AEU, arbitrary erythema units; TAS, total antioxidant stress; MDA, malondialdehyde; BMI, body mass index.

## Discussion

Rubeosis faciei diabeticorum is a cutaneous feature of diabetes mellitus that manifests as a facial erythema. Even though there are no studies that have actually investigated it, there was a widely accepted belief that it is a form of diabetic microangiopathy.^10^ The results of this study contradict this belief.

Hyperglycemia and accumulation of AGEs promotes development of microvascular diabetic complications.^16^, ^19^ Examination of skin biopsy specimens has demonstrated that accumulation of AGEs in the skin is associated with the presence and severity of microvascular diabetic complications. In prospective studies, the levels of AGEs in the cutaneous biopsy samples could even predict the risk of developing diabetic retinopathy. Skin autofluorescence is a non-invasive, validated method of estimating AGE levels in the skin. A systemic review demonstrated an association between skin autofluorescence and the development of diabetic microvascular complications.^19^ It was expected that patients with rubeosis faciei diabeticorum would have more pronounced hyperglycemia and accumulation of cutaneous AGEs. However, in the present study, the facial erythema index did not correlate with either HbA1c or skin autofluorescence.

Oxidative stress is considered as one of the factors that links hyperglycemia to the development of microangiopathies.^15^, ^16^ A plethora of studies have acknowledged increased oxidative stress in patients with diabetes mellitus, with a further aggravation of oxidative stress in patients with diabetic retinopathy, neuropathy, and nephropathy.^28^, ^29^ It seemed plausible to assume that oxidative stress plays an important role in the development of rubeosis faciei diabeticorum.

MDA is a marker of lipid peroxidation, namely, in the event of oxidative stress. MDA has been previously reported to be elevated in cases of diabetes.^30^ Patients with microvascular diabetic complications tend to have higher MDA levels then patients with uncomplicated diabetes, as measured by thiobarbituric acid reactive substances (TBARS) assay.^31^ This has been particularly observed in diabetic patients with neuropathy and chronic non-healing wounds, as well as in cases of diabetic retinopathy.^28^, ^32^

In the present study there was no significant correlation between MDA levels and the degree of facial erythema. It should be noted that chromatographic assays are considered the gold standard for the determination of MDA levels in plasma because of their improved specificity. However, this method is expensive and burdensome, therefore TBARS assay is still widely used.^33^

Blood plasma contains various compounds with antioxidant properties, including bilirubin, uric acid, ascorbic acid, polyphenols, and protein thiol groups. It is technically difficult to measure the contribution of each antioxidant separately. Therefore, TAS is a convenient method to describe the overall antioxidant capacity of known and unknown antioxidants in plasma, taking into account their synergistic and antagonistic affects. The limitation of TAS assay is that it does not measure the contribution of antioxidant enzymes to the antioxidant capacity of plasma.^25^, ^34^, ^35^, ^36^

In the present study, facial erythema in diabetic patients correlated positively with TAS. This also contradicts the authors’ expectations. TAS levels have previously been reported to be decreased in patients with diabetes mellitus.^37^, ^38^ Lower TAS levels have been noted in patients with diabetic retinopathy in comparison to uncomplicated diabetes.^39^ Authors usually interpret this as a sign of oxidative stress. Uncommonly, increased TAS level in diabetes mellitus has also been described.^40^, ^41^ TAS changes can be mediated by several factors, thus results should always be interpreted with caution.^25^, ^34^, ^35^, ^36^ For instance, bilirubin has been known to elevate TAS levels.^42^ Bilirubin functions as an endogenous antioxidant and also promotes angiogenesis *via* VEGF. Clinically raised serum bilirubin levels in diabetic patients have been associated with a lower risk for the development of a diabetic foot and for lower-limb amputation.^37^, ^43^ *In vivo* experiments on diabetic rats have demonstrated enhanced wound healing upon an application of an ointment containing bilirubin. In this case, bilirubin promoted angiogenesis in the diabetic wound by upregulating the expression of VEGF.^44^ Additionally, increased serum uric acid levels can also influence TAS, and have been linked to a higher risk of developing diabetic retinopathy.^25^, ^34^, ^35^, ^36^, ^45^ Theoretically, both uric acid and bilirubin can cause TAS elevation, as well as promote the development of rubeosis faciei diabeticorum. Unfortunately, these parameters were not assessed in the current investigation. It is advisable to measure bilirubin and uric acid levels, and examine their association with the development of the facial erythema in diabetic patients.

## Conclusions

Rubeosis diabeticorum faciei was not associated with elevated levels of HbA1c, skin autofluorescence, and MDA. However, there was a significant positive correlation with TAS. This contradicts the original expectations. Elevation of TAS could have been caused by several factors that might have also influenced development of rubeosis faciei, including hyperbilirubinemia and hyperuricemia. This hypothesis should be tested in further studies. Direct association between rubeosis diabeticorum faciei and diabetic retinopathy should also be investigated.

## Author's contribution

Aleksejs Zavorins: Statistical analysis; approval of the final version of the manuscript; conception and planning of the study; elaboration and writing of the manuscript; obtaining, analyzing and interpreting the data; effective participation in research orientation; intellectual participation in propaedeutic and/or therapeutic conduct of the cases studied; critical review of the literature; critical review of the manuscript.

Alise Silova: Approval of the final version of the manuscript; conception and planning of the study; obtaining, analyzing and interpreting the data; effective participation in research orientation; intellectual participation in propaedeutic and/or therapeutic conduct of the cases studied; critical review of the literature; critical review of the manuscript.

Julija Voicehovska: Approval of the final version of the manuscript; conception and planning of the study; elaboration and writing of the manuscript; obtaining, analyzing and interpreting the data; effective participation in research orientation; intellectual participation in propaedeutic and/or therapeutic conduct of the cases studied; critical review of the literature; critical review of the manuscript.

Janis Kisis: Approval of the final version of the manuscript; conception and planning of the study; obtaining, analyzing and interpreting the data; effective participation in research orientation; intellectual participation in propaedeutic and/or therapeutic conduct of the cases studied; critical review of the literature; critical review of the manuscript.

## Financial support

Riga Stradins University, Doctoral Studies Grant.

## Conflicts of interest

None declared.
